# Anthocyanin Hybrid Nanopigments from Pomegranate Waste: Colour, Thermomechanical Stability and Environmental Impact of Polyester-Based Bionanocomposites

**DOI:** 10.3390/polym13121966

**Published:** 2021-06-14

**Authors:** Bàrbara Micó-Vicent, Marina Ramos, Valentin Viqueira, Francesca Luzi, Franco Dominici, Andrea Terenzi, Etienne Maron, Mahmoud Hamzaoui, Stephane Kohnen, Luigi Torre, Alfonso Jiménez, Debora Puglia, María Carmen Garrigós

**Affiliations:** 1 Colour and Vision Group, University of Alicante, San Vicente del Raspeig, ES-03690 Alicante, Spain; barbara.mico@ua.es (B.M.-V.); valentin.viqueira@ua.es (V.V.); 2 Department of Applied Statistics and Operational Research, and Quality, Universitat Politècnica de València, ES-03801 Valencia, Spain; 3 Department of Analytical Chemistry, Nutrition & Food Sciences, University of Alicante, San Vicente del Raspeig, ES-03690 Alicante, Spain; marina.ramos@ua.es (M.R.); alfjimenez@ua.es (A.J.); 4 Department of Civil and Environmental Engineering, University of Perugia, 05100 Terni, Italy; francesca.luzi@unipg.it (F.L.); francodominici1@gmail.com (F.D.); andrea.terenzi@unipg.it (A.T.); luigi.torre@unipg.it (L.T.); 5 Biomass Valorisation Platform, Celabor scrl, Avenue du Parc 38, 4650 Herve, Belgium; etienne.maron@celabor.be (E.M.); mahmoud.hamzaoui@celabor.be (M.H.); stephane.kohnen@celabor.be (S.K.)

**Keywords:** pomegranate waste, anthocyanins, natural hybrid nanopigments, nanoclays, bionanocomposites, life cycle analysis

## Abstract

In the present work, anthocyanin (ACN) hybrid nanopigments were synthetized by using a natural pomegranate dye (PD) and calcined hydrotalcite (HT) and montmorillonite (MMT) nanoclays. A wide colour gamut was obtained with MMT-based nanopigments ranging from reddish to bluish hues caused by structural transformations of ACNs at different pH values. However, a buffer effect was observed with HT obtaining samples a similar final colour regardless of the synthesis conditions. Nanopigments added with a biomordant extracted from pomegranate peels showed a different colour compared to the incorporation of a commercial mordant due to the intrinsic colouring properties of the pomegranate bioadditive. The developed nanopigments were incorporated at 7 wt% loading to produce novel polyester-based bionanocomposites which were characterized in terms of thermal, mechanical and colour properties. The encapsulation of PD into the nanoclays improved its thermal stability, in particular for MMT-based nanopigments. The pH changes observed during the nanofillers synthesis affected the final colour of the MMT-based nanocomposites, inducing a general increase in ∆E* and a decrease in gloss values. Slight improvements were obtained in terms of elastic modulus for MMT-based polymer samples confirming the applicability of the developed bionanocomposites as colouring and reinforcement materials. A very similar environmental profile was obtained for MMT and HT-based nanofillers showing MMT-based nanopigments a slightly better general behaviour. The results of the LCA study evidenced the suitability of the processes used in this work to the circular bioeconomy approach through sustainable food waste management and the production of bioplastics using waste substrates.

## 1. Introduction

In the last decades, dyes/pigments and clays were largely used and applied to create coloured design materials. The prospective and application of natural dyes and pigments extracted from natural sources (fruits, vegetables and in general from natural wastes) [1,2] represent an eco-friendly and non-toxic strategy to limit the use of chemical-based products. The environmental problems related to chemical pollution have encouraged the replacement of synthetic dyes by natural extracts, which exhibit better biodegradability and are able to minimize the health hazardous problems and negative aspects related to genotoxicity, cytotoxicity and carcinogenicity of synthetic dyes [3,4,5]. Polyphenolic dyes extracted from plant leaves and pomegranate rind were used as an alternative eco-friendly approach for nylon dyeing [6]. Natural pigments were also added to polymer matrices not only for colouring purposes, but even for their deodorant, antioxidant and antimicrobial properties [7]. Mitrae et al. proposed the use of tomato-based pigments as colouring agents in poly(vinyl alcohol)-based films [1]. Other studies also focused on the dyeing properties of polypropylene and polyamide-based systems prepared by melt compounding the polymer with six-layered clays. Razafimahefa et al. found that the introduction of nanoclays improved the dyeing ability of nylon added with dispersed dyes [8,9] and they proposed the production of dyeable polypropylene fibres added with nanoclay particles in the polymer matrix.

The dyes responsible for different colours such as blues, reds and purples in many flowers, fruits and vegetables are members of a subclass of flavonoid colorants known as anthocyanins (ACNs). ACNs present great antioxidant activity against many chronic diseases and numerous studies reporting their nutritional, medicinal and therapeutic value can be found [10,11,12]. These compounds can be used as natural food colorants and pH indicators in aqueous solutions due to some modifications in their structure, undergoing a variety of molecular rearrangements with changes in pH, resulting in colour changes from blue to red [13,14]. This behaviour is mainly attributed to the number of hydroxyl and methyl groups present in the ACN molecular structure and these colour changes are the result of the rearrangement of one compound, unlike universal indicators, which use several different compounds to get the same effect [15]. Intelligent pH indicative polymer films containing ACNs extracted from different wastes, such as red cabbage [16] and rose [17] were produced. However, the colour and stability of ACNs are mainly influenced by pH, co-pigmentation and solvent effects, temperature, oxygen, light and structure [18]. Therefore, ACNs are unstable, and they can suffer chemical transformations depending on the medium conditions which could limit their wide applications. The development of stabilization strategies to improve the colour stability of ACNs such as the addition of co-pigments, formation of supramolecular complexes and encapsulation systems, and protection with natural clay minerals were proposed by Li et al. [19]. In this sense, it has been proved that colour stabilization can be achieved by using hybridization of clay nanostructures [20,21,22]. A number of recent studies have focused on the adsorption of ACNs to obtain dye/clay hybrid pigments with improved colour fastness and stability which include reports of stable hybrid pigments prepared from ACNs and several clay minerals that undergo reversible pH-dependent colour changes [23,24,25]. Li et al. [21] incorporated ACNs extracted from fresh blue berry fruits into different clay minerals including tubular halloysite, fibrous sepiolite, lamellar kaolinite and montmorillonite to obtain reversible allochronic hybrid pigments. Lima et al. [26] developed hybrid pigments based on organo-saponite (cetyltrimethylammonium bromide) and ACNs from red grape. They observed a stabilization of the different chemical structures of ACNs (blue quinoidal base form and red flavylium cation), exhibiting the resulting hybrid reversible acid/base chromism from blue to red and vice versa. In addition, saponite showed a cation exchange mechanism that led to the intercalation of the flavylium cation of the natural dye as well as intermolecular interactions in the stabilization of the quinoidal base form.

Pomegranate (*Punica granatum L.*) is a well-known edible fruit widely consumed as fresh fruit and juice and it is considered a ‘super fruit’ since it is rich in antioxidant phytochemicals, being recognized for a myriad of health benefits [27,28,29]. Moreover, the juice is gaining popularity worldwide for its uniqueness, exclusive colour, taste and high content in phytochemicals, mainly polyphenols. Most studies on pomegranate have focused on polyphenols, flavonoids, hydrolysable tannins (ellagitannins), anthocyanins, anthocyanin−flavanol and flavanol−anthocyanin adducts, and proanthocyanidins. Among the ACNs pigments, cyanidin-3-glucoside is the major anthocyanin found in most of the plants, particularly in pomegranate juice [14].

Surfactants, mordant salts and silane agents (as well as the combination of these components) have been used in nanoclay structures to obtain high-performing hybrid nanopigments. These modifiers can open the laminar nanoclay structures and improve their exchange and adsorption dye (dyeability) capacities. The use of design of experiments (DoE) has been also considered to find the best combination of modifiers in nanopigments synthesis to maximize the adsorption dye capacity and the thermal stability of the natural dyes, in view of their application as polymer additives in biopolyester-based formulations, obtaining acceptable thermal, mechanical and colour stability performance [30]. Regarding mordant salts, new plant species have been proposed as a source of sustainable metal salt-free biomordants to replace metallic and toxic mordants. Rather et al. [31] and Ul-Islam et al. [32] reported an improvement in wool dyeing when using natural tannin mordants extracted from pomegranate peel, babool bark, catechu and gallnut resulting in a broad colour spectrum an acceptable colour strength and fastness properties. In our work, tannins extracted from pomegranate peels were used in order to test the effectiveness of this biomordant obtained from the same waste.

The research in hybrid materials based on the use of clay minerals and natural pigments has continuously increased during the last decades [33,34,35]. ACNs were used in food packaging applications for the development of intelligent films with reversible acid/base allochronic behaviour [23], new colorimetric films based on pH using mulberry ACNs, ethylene-vinyl alcohol copolymer and montmorillonite [33]; intelligent nanocomposite films based on chitosan and poly-vinyl alcohol with ACNs from black carrot [36] or active intelligent nanocomposite films based on ethylene vinyl acetate containing ACNs from rosemary extract and ZnO/Fe-MMT nanoparticles [37]. Therefore, the wide palette of ACNs colours combined with the stability offered by clay minerals have improved the applicability of the obtained hybrid pigments in sectors such as food packaging and textile fabrics [38]. Despite the wide use of modified clays to enhance the stability of organic dyes, a minimal number of works have studied the synthesis of hybrid nanopigments by using design of experiments. In addition, to the best of our knowledge, the stability enhancement of natural pomegranate waste pigments at different pH values on modified clays and the further incorporation of the synthetized hybrid pigments in polymer materials have not been explored yet.

The aim of this work was the incorporation of a pomegranate dye, rich in ACNs and extracted from agricultural fruit wastes, into different nanoclay structures (MMT, HT) to obtain anthocyanin hybrid nanopigments to be used as biopolymer colouring and reinforcing additives. The interactions between ACNs and nanoclays were also investigated in terms of colour behaviour with pH. The possibility of replacing a traditional commercial mordant salt by a biomordant obtained from pomegranate peels was also studied. Novel polyester-based bionanocomposites containing 7 wt% of the hybrid nanofillers were developed and characterized in terms of thermal, mechanical and colour properties. Life cycle analysis (LCA) was considered to verify the environmental impact of the selected procedures for clay functionalization as a function of clay typology. Differences in environmental profiles were also evaluated for polymer materials obtained by using the commercial polyester matrix and for the different developed nanopigments.

## 2. Materials and Methods

### 2.1. Materials and Reagents

Pomegranate waste was obtained from discarded whole fruits from FECOAM (Murcia, Spain). Pomegranate fruits were cleaned, and the husk was then removed manually in order to obtain pomegranate peels. A pomegranate natural pigment rich in ACNs was obtained from pomegranate juice and supplied by Celabor scrl (Herve, Belgium).

Two laminar nanoclays with different ion exchange properties, anionic hydrotalcite (HT) and cationic montmorillonite (MMT), were selected in this work based on previous research with laminar nanoclays and the enhanced properties found in different polymer matrices by achieving laminar nanoclay exfoliation [39]. Hydrotalcite (HT, BioUltra, ≥99.0%) and montmorillonite (MMT, Gel White) were supplied by Sigma-Aldrich (St. Louis, MO, USA) and Southern Clay Products (Gonzales, TX, USA), respectively. HT was calcined at 600 °C for 3 h before use. Sodium dodecyl sulphate (SDS) and cetylpyridinium bromide (CPB) were used as surfactants for HT and MMT, respectively. Aluminium potassium sulphate dodecahydrate and (3-Aminopropyl) triethoxysilane were also used as mordant salt and coupling agent modifiers, respectively. All reagents and chemicals were of analytical grade, and they were purchased from Sigma-Aldrich (St. Louis, MO, USA).

For bionanocomposites preparation, INZEAF2 biopolyester commercial grade (moisture content <0.5%, density of 1.23 g cm^−3^ measured at 23 °C, melt flow rate of 19 g/10 min (2.16 kg, 190 °C)) was kindly supplied by Nurel (Zaragoza, Spain).

### 2.2. UPLC-DAD-MS Analysis of Pomegranate Natural Pigment

The identification and quantification of the major ACNs present in the pomegranate natural pigment was performed, in triplicate, by UPLC-DAD-MS. A Waters UPLC system coupled to a Xevo TQ-S detector (Waters Corporation, Milford, MA, USA) was used. Chromatographic separation of ACNs was performed with an ACQUITY UPLC Shield RP18 column (100 mm × 2.1 mm I.D. × 1.7 µm) (Waters Corporation, Milford, MA, USA) at 40 °C and a flow rate of 0.5 mL min^−1^. Then, 20 mg of pigment were dissolved in 5 mL of MeOH:formic acid 0.1% (*v*/*v*) in an ultrasonic bath for 15 min and passed through a 0.22 µm poly(tetrafluoroethylene) (PTFE) filter. The mobile phase was composed of ultrapure water containing 0.6 wt% formic acid and 126 mg ammonium formate (A) and acetonitrile (B). The linear gradient used was: 1% B to 21% B (5 min) to 100% B (4 min), 100% B (2 min), 100% B to 1% B (1.5 min) and 1% B (3.5 min). A total of 2 μL of sample was injected and UV–Vis chromatograms were recorded at 280 nm. Spectra were acquired in the negative ionization mode. Desolvation was carried out with a N_2_ gas flow of 800 L h^−1^ and 550 °C. Cone voltage was set at 2 kV. Specific multiple reaction monitoring (MRMs), cone and collision voltages were used for each compound, as detailed in Table 2. Quantification of major ACNs was performed based on integrated peak areas of samples and standards diluted in MeOH:formic acid 0.1% (*v*/*v*) using external calibration (1–10 mg kg^−1^).

### 2.3. Pomegranate Biomordant Extraction

The biomordant used in this work was obtained by microwave-assisted extraction (MAE) using a FLEXIWAVE™ microwave oven (Milestone srl, Bergamo, Italy) [40]. Pomegranate peels were dried at 40 °C for 24 h in a climatic chamber (Dycometal, Barcelona, Spain) at 25% of relative humidity (RH). Dried peels were ground with a high-speed rotor mill at 12,000 rpm (Ultra Centrifugal Mill ZM 200, RETSCH, Haan, Germany) to obtain a powder of 0.5 mm of particle size. Then, MAE was carried out with 6 g of sample mixed with 60 mL of 40% (*v*/*v*) ethanol for 10 min at 65 °C. The obtained biomordant extract was filtered and purified by adding 96% (*v*/*v*) ethanol at −20 °C overnight to precipitate polysaccharide compounds. After that, the sample was vacuum-filtered and the solvent was removed by using a rotary evaporator (R-300, Büchi Labortechnik AG, Switzerland). The final aqueous solution was freeze-dried (LyoQuest Plus, Telstar, Terrassa, Spain) and the purified biomordant was stored in the darkness at room temperature until further use.

### 2.4. Synthesis of Pomegranate Hybrid Nanopigments (PDNPs)

The synthesis of pomegranate hybrid nanopigments (PDNPs) was performed by using the water/organic solvent dispersion method, based on previous works [7]. For the synthesis of MMT-based PDNPs, mechanical stirring at 1800 rpm for 24 h in distilled water was performed to ensure the maximum nanoclay (25 g L^−1^) swelling. The different pH values were reached by using an aqueous HCl solution (2 mL L^−1^). The pomegranate dye (PD) was added after the preparation of a high dye concentration solution in distilled water. The PD dispersion was carried out at 1800 rpm for 20 h after the dye solution addition. After that, the dispersion speed was reduced to 600 rpm for 4 h as an aging step. The addition of surfactant and/or mordant additives was performed before the dye addition, while silane was added after the dye exchange. The whole process was carried out at room temperature. Solvent separation was performed by centrifugation obtaining a paste-like nanopigment which was finally freeze-dried for 24 h. A similar procedure was used for HT-based PDNPs but using 50% (*v*/*v*) ethanol as solvent for nanoclay dispersion.

For MMT-based PDNPs, a first set of experiments was designed to combine MMT nanoclay with pH changes and the presence of different modifiers (commercial mordant salt, surfactant or silane coupling agent). The total amount of pomegranate dye (PD) loaded for each sample was 0.56 g L^−1^ and samples were coded as ATM1-ATM12. After that, the natural pH of MMT was fixed and colour changes under different modifiers concentration and using a higher PD content were considered (samples ATM13-ATM18). An additional experiment was performed by replacing the commercial mordant present in ATM14 formulation by the biomordant obtained from pomegranate peels (sample ATM19) in order to study the influence of using this biomordant on the nanopigment synthesis. All the other steps remained unaltered.

In the case of HT-based PDNPs, only four samples were obtained (ATH1-ATH4) as it was observed that HT nanoclay acted like a buffer and so it was not possible to change the pH values during the exchange step, regardless of the quantity of added HCl, obtaining a similar final colour. All conditions used for the synthesis of MMT and HT-based PDNPs are shown in Table 1.

### 2.5. Bionanocomposites Preparation

Bionanocomposites based on INZEAF2 were obtained by melt blending the biopolymer and the synthetized pomegranate-hybrid additives at 7 wt%. A co-rotating twin-screw extruder, Xplore 5 and 15 Micro Compounder by DSM (Xplore Instruments, Geleen, The Netherlands), was used by mixing at a rotating speed of 90 rpm for 3 min, setting a temperature profile of 190–195–200 °C in the three heating zones from feeding section to die. A Micro Injection Moulding Machine 10cc by DSM (Xplore Instruments, Geleen, The Netherlands), coupled to the extruder and equipped with adequate moulds, was used to produce samples for tensile tests according to the standards. An appropriate pressure/time profile was used for the injection of each type of samples, while the temperatures of the injection barrel and the moulds were set, respectively, at 210 and 30 °C.

### 2.6. Bionanocomposites Characterization

Thermal degradation of pomegranate hybrid nanopigments (PDNPs) and bionanocomposites was evaluated by thermogravimetric analysis (TGA, Seiko Exstar 6300, Tokyo, Japan). Around 5 mg of samples were heated from 30 to 900 °C at 10 °C min^−1^ under nitrogen atmosphere (200 mL min^−1^).

Tensile tests were carried out for bionanocomposites with a universal test machine LK30 (Lloyd Instruments Ltd., Bognor Regis, UK) at room temperature following the ASTM D638–14 standard. Five replicates were performed using a 5 kN load cell and setting the crosshead speed to 5 mm min^−1^. The values of strength at break (σ_b_), strain at break (ε_b_) and Young Modulus (E) were collected as tensile parameters.

Optical properties of bionanocomposites were studied with a Konica Minolta sphere integrated spectrophotometer (CM-2300d, Tokyo, Japan). Data were acquired by using the SCI 10/D65 method and CIELAB colour variables were used. Samples were placed on a white standard plate and L*, a*, and b* parameters were determined. Measurements were performed, in triplicate, at random locations on each sample. Total colour difference ΔE*, chroma and gloss values were calculated.

### 2.7. Life Cycle Assessment (LCA)

The life cycle assessment (LCA) of the produced polymer samples was performed following the ISO 14040 and 14044 standards, by using an attributional approach. The main aim of this analysis was to compare the effect, in terms of materials and energy consumption and related environmental impacts, of functionalizing both MMT and HT-based PDNPs and their mixing with a commercial biopolyester. The materials were analysed by considering a “from cradle to gate” approach; therefore, the use phase and the end of life of final products were not considered in this work. Essentially, three main processes were analysed and assembled to calculate the overall environmental performance of the products: (i) extraction of functional molecules from pomegranate waste, (ii) MMT and HT functionalization with PD and (iii) polyester-based compounding production by melt blending. The life cycle impact assessment was performed by considering ReCiPe 2016 and IPCC 20yr. methodologies. The study was implemented by using SimaPro 8.5 software (PRé Sustainability, Amersfoortm, The Netherlands) and Ecoinvent 3.5 libraries (Ecoinvent, Zurich, Switzerland). Primary data were used for all inventory parameters directly connected to the production process, whereas the secondary data (libraries) were used for raw materials and used energy sources.

### 2.8. Statistical Analysis

Average data from replicate determinations were reported along with standard deviation values. Analysis of Variance (ANOVA) and Tukey’s honest significant difference test were performed at *p* < 0.05. Statistical analyses were carried out using Statgraphics Centurion XVI (Version 16.1.11, StatPoint Technologies Inc., Warrenton, VA, USA).

## 3. Results

### 3.1. Characterisation of Pomegranate Natural Pigment

**Scheme 1:**
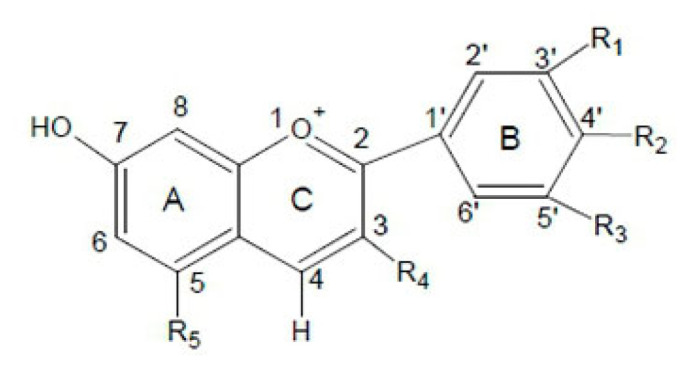
Structure of major ACNs found in pomegranate pigment.

Table 2 summarizes the major anthocyanins identified and quantified in the pomegranate natural pigment, including delphinidin-3-glucoside (Dp-3-glu), cyanidin-3-glucoside (Cy-3-glu), pelargonidin-3-glucoside (Pg-3-glu), delphinidin-3,5-diglucoside (Dp-3,5-diglu), cyanidin-3,5-diglucoside (Cy-3,5-diglu) and pelargonidin-3,5-diglucoside (Pg-3,5-diglu). Cy-3,5-diglu was the predominant ACN followed by Dp-3,5-diglu, accounting for 84.4% of the total fraction, representing ACN diglucosides 24.2% of the pigment. The general chemical structure of the six anthocyanins identified in the pomegranate pigment is reported in Scheme 1. The substitution of the B-ring with OH groups tends to increase the maximum absorption of ACNs, altering their colour. The intense red colour of pomegranate juice has been reported to be due to the presence of anthocyanins, mainly 3-glucosides and 3,5-diglucosides of cyanidin (red pigments), delphinidin (purple pigments), and pelargonidin (orange pigments) [27,41], in agreement with the results found in this work. Turfan et al. [42] also detected the same six ACNs shown in Table 2 in the pomegranate juice of several pomegranate cultivars from different geographical regions, being the relative amounts of ACNs dependent on variety, climatic and cultural variables.

ACNs are generally identified by their flavylium cationic forms (Table 2) which are related to the final colour observed for ACNs in aqueous solution, due to a series of reactions that take place with thermodynamic and kinetic implications, mainly based on different pH values and equilibrium constants [10,43]. ACNs act as weak diacids and they become deprotonated with increasing pH values, increasing maximum absorption towards higher wavelengths. The pH-dependent dynamic equilibrium of the different ACN structural forms present in aqueous solutions was reported by Fedenko et al. [15] and Houghton et al. [44]. The red flavylium cation (AH^+^) is favoured at very acid pH values (pH < 2–3), whereas at higher pH values some reversible (to some extent) chemical transformations could occur. At pH values higher than pKa_1_, the deprotonation of AH^+^ gives the neutral quinoidal base (A) which is characterized by purple tonalities. At pH values above pKa_2_, A is further deprotonated obtaining the blue anionic quinoidal base form (A^−^). These two subsequent deprotonations result in a shift in maximum wavelength of 20–30 nm and 50–60 nm for A and A^−^, respectively. Due to the instability of A and A^−^ forms, the transformation of AH^+^ to A is normally less thermodynamically favourable than the hydration of AH^+^ forming the colourless hemiketal form B. This reaction breaks the ring’s aromaticity and produces the loss of adsorption properties, transforming the initial structure into an uncoloured carbinol-pseudo base. Finally, the B form transforms tautomerically by ring opening into yellow *cis*-chalcone C_c_ and isomerizes to *trans*-chalcone C_T_.

**Figure 1:**
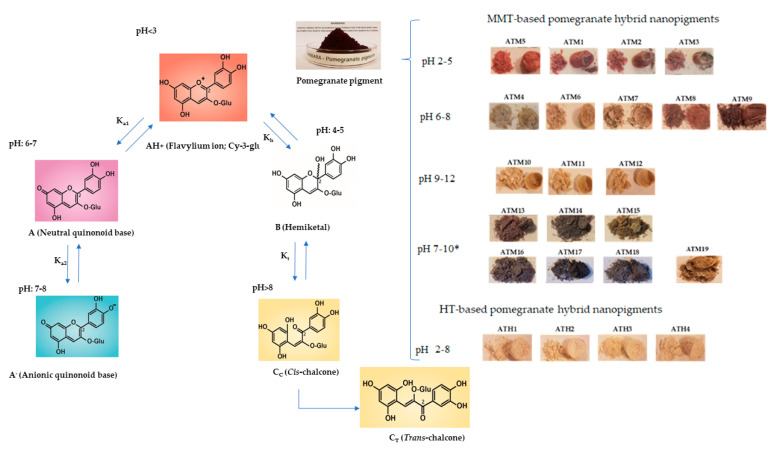
Structural modifications as a function of pH and colours obtained during the synthesis of pomegranate hybrid nanopigments (PDNPs) (adapted from [44]).

According to this behaviour, the final ACN colour will be the result of the ratio of these different species that will be in equilibrium at a particular pH value. It has been reported that at least four species, apart from the flavylium cation, could be present in solution at moderately acid conditions, constituting a pH reversible network of chemical reactions. In addition, the colour given by ACNs is also function of their concentration due to self-aggregation which takes place at relatively low ACNs concentrations [43]. Figure 1 shows a scheme of the different chemical reactions than could occur for any ACN flavylium derivative, in general [29].

### 3.2. Pomegranate Hybrid Nanopigments (PDNPs)

Currently, one of the most promising strategies for stabilizing the colour of ACNs and flavylium cations is their encapsulation by intercalating and/or adsorbing them on solid surfaces and/or in confined spaces [24]. In particular, the colour stability of ACNs is very limited under alkaline conditions which limits their practical use [45]. The visual aspect of the colours obtained for the different synthetized hybrid nanopigments is included in Figure 1. As it can be seen, in the case of MMT-based PDNPs a wide palette of colours ranging from red to blue hues were obtained. This behaviour could be related to the different pH values used in the synthesis conditions (Table 1) and previous discussion in Section 3.1 related to the main ACNs present in the pomegranate pigment and their stability with pH values. In addition, the colour of the clay minerals, in particular MMT, could also affect the colour of the resulting hybrid nanopigments [21].

As it is shown in Figure 1, reddish nanopigments were obtained for samples ATM1-ATM3 and ATM5, with initial pH values ranging from 2–5 and final pH values ≤ 7 (Table 1), suggesting a good stabilization of PD in its structural flavylium cation form AH^+^, by its intercalation into MMT and the important electrostatic interaction between the dye and the silicate layers [45]. In particular, the most intense red colour was observed for ATM5 in accordance with pH initial and final values, being AH^+^ the predominant form [24]. Regarding the other nanopigments, their final colour is expected to be a combination of flavylium cation form and quinoidal base formation at synthesis conditions, considering that these forms will be in equilibrium at the studied pH values as the acid-base reaction is the fastest of the network of chemical reactions [43].

Increasing pH values at synthesis conditions (Table 1) lead to structural transformations of ACN molecules loaded on the nanoclay. In this sense, at moderately acid-neutral conditions the flavylium and base forms could be in fast equilibrium while AH^+^ is reacting to give the hemiacetal form B followed by ring-opening tautomerization to form the *cis*-chalcone form and then slow isomerization to the *trans*-chalcone form; considering that hydration is much slower compared to proton transfer [24,35,43], resulting in orange to purple hues (Figure 1). At basic pH values, yellowish or bluish colours were obtained as ACNs are prone to colour changes due to both nucleophilic attack by water and deprotonation. These colour changes could be attributed also to the different number of –OH and methoxy groups present that affected conjugated double bonds of the ACN skeleton [21]. The presence of surfactant, mordant and silane modifiers (Table 1), at the same initial pH values, could also modify the nanoclay surface polarity and interlayer space as well as the electrostatic interactions between MMT and ACN dyes, influencing PD adsorption and its stabilization [2]. In addition, the use of higher amounts of PD in samples ATM13-ATM18 could also affect its intercalation into the interlayer space of MMT and facilitate or inhibit the attack of hydroxyl ions to the ACN dye under alkaline conditions due to steric hindrance effects [45].

Regarding HT-based PDNPs, it should be considered that HT exhibits anion exchange properties and its ability to host anionic dyes has been reported by different authors [2]. So, cationic dyes will be well stabilized by its intercalation into the layers of cation exchangeable clays, such as MMT, and the stability of naturally occurring ACN dyes has been reported to be greatly enhanced by their complexation with MMT [46]. As a result, under the tested experimental conditions (Table 1), the cationic flavylium structure in ACNs was not able to be stabilized into HT obtaining a similar yellowish final colour for all synthetized nanopigments, suggesting the predominant formation of chalcone forms, as already explained.

**Figure 2:**
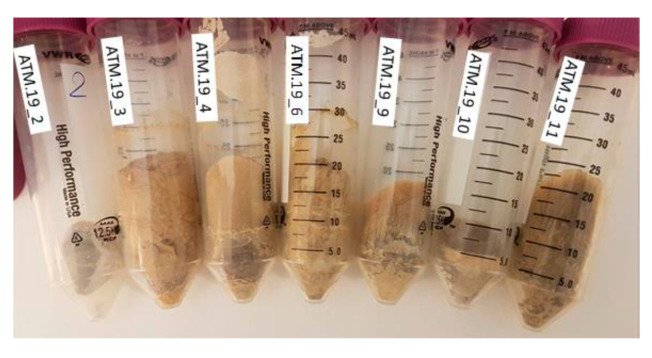
ATM19-based PDNPs obtained by using synthesis conditions shown in Table 3.

Finally, concerning the biomordant-based hybrid nanopigment ATM19, the final colour of this sample was significantly different from the equivalent one containing the commercial mordant (ATM14). This behaviour was essentially attributed to the intrinsic orange-brownish colour of the biomordant additive compared to the whitish colour of the commercial mordant. The own colour properties of pomegranate peel biomordant to act itself as a source of colour for dyeing and the possible synergistic effects on shades when being used in conjunction, at different concentration levels, with other dyes were reported by Singhee D. [47]. Different additional experiments were carried out with the aim of studying the effect of decreasing the biomordant concentration and using different concentrations of CPB and silane modifiers (ranging from 1–5 wt%). However, all the obtained samples showed a similar brownish colour regardless of their additives’ composition (Figure 2 and Table 3).

#### Thermal Stability of PDNPs

**Figure 3:**
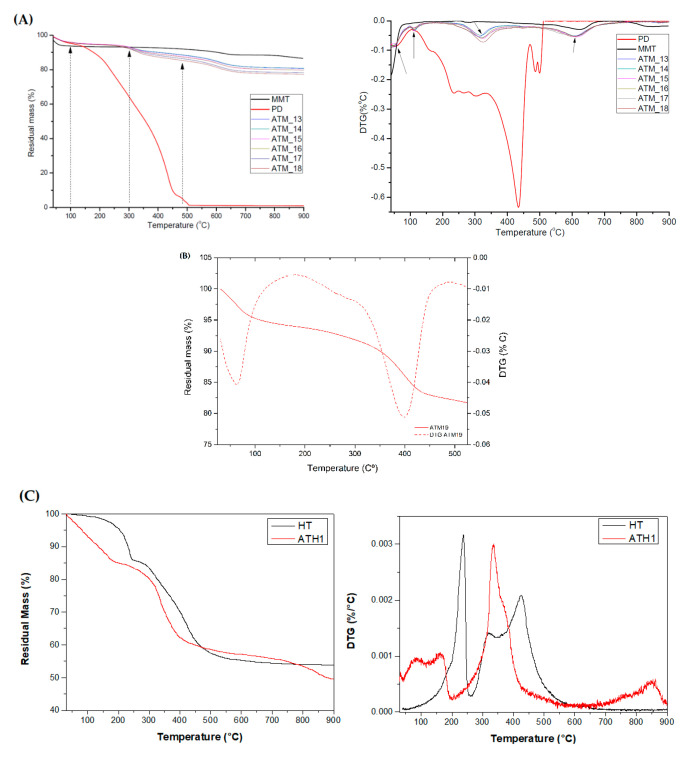
TG and DTG results obtained for (**A**) MMT, PD, and ATM13-ATM18 PDNPs; (**B**) zoomed area for ATM19; (**C**) HT, PD, and ATH1.

Natural ACNs have been reported to be highly unstable with temperature and they are susceptible to various degradation reactions [48]. TG and DTG curves of PD and PDNPs were performed in order to study their overall thermal stability. Figure 3 shows the results obtained for PD, nanoclays (MMT, HT) and ATM13-ATM19 and ATH1 hybrid samples, as an example (similar results were obtained for the rest of the developed MMT and HT-based PDNPs). In the case of PD, different thermal events were observed with peak temperatures ranging between 100 and 235 °C, which were associated to the loss of surface water and volatile low molar mass components of the dye (approximately 12 wt% of the initial mass) [49]. Other losses were also observed at temperatures around 235 °C and 440 °C, which were probably related to the loss of organic material and thermal decomposition of the different components present in PD [50] which were degraded to phenolic acids and aldehydes by deglycosylation and ring opening reactions [51]. This behaviour indicates a poor high temperature resistance for the studied natural pigment. Regarding hybrid nanopigments, temperatures below 200 °C were due only to structural water losses. A higher thermal stability was observed for the synthetized ATM-based hybrid nanopigments, not showing a significant thermal degradation until approximately 315 °C (which was related to the decomposition of PD) compared to that observed for PD at 160 °C, significantly improving and protecting PD degradation at high temperature processing conditions. The final degradation corresponding to MMT clay was observed around 600 °C [52]. Different residual weight loss values were obtained at the end of the test (900 °C) for ATM-based samples containing different initial PD content. So, a higher weigh loss corresponding to the maximum dye loading was obtained for sample ATM17 whereas comparable and lower residual weight values were observed for ATM13-ATM16 and ATM18 samples (with 1.11 gL^−1^ PD loading). The stability of the ATM19 hybrid nanopigment was lower compared to ATM-based samples containing the commercial mordant and this behaviour was related to the own degradation of the added biomordant.

Regarding HT, two main degradation steps were observed for this nanoclay, according to the literature, corresponding to the decomposition of structural hydroxyl groups and the decomposition of interlayer carbonate anions around 230 °C and 440 °C, respectively [53]. Concerning ATH-based hybrid nanopigments, a different degradation behaviour was observed compared to neat HT due to PD addition. The PD decomposition in ATH1 was observed at higher temperatures compared to PD alone, confirming the stabilisation effect due to PD intercalation in HT [54]. However, a lower thermal stabilization was observed compared to ATM-based samples (Figure 3), providing MMT nanoclay better thermal stability and encapsulation properties of ACNs than HT.

### 3.3. Bionanocomposites Characterization

The obtained MMT and HT-based pomegranate hybrid nanopigments were added at 7 wt% loading into INZEA biopolymer and the developed bionanocomposites were characterized in terms of colour, thermal, and mechanical properties. Blank samples of neat MMT and HT nanoclays were also extruded and used as control. The proposed weight amount was selected according to previous works where, regardless of the final colour of the samples, an enhancement in mechanical properties was obtained without limiting the agglomeration of the nanoscale additives [2].

#### 3.3.1. Colour Properties

**Figure 4:**
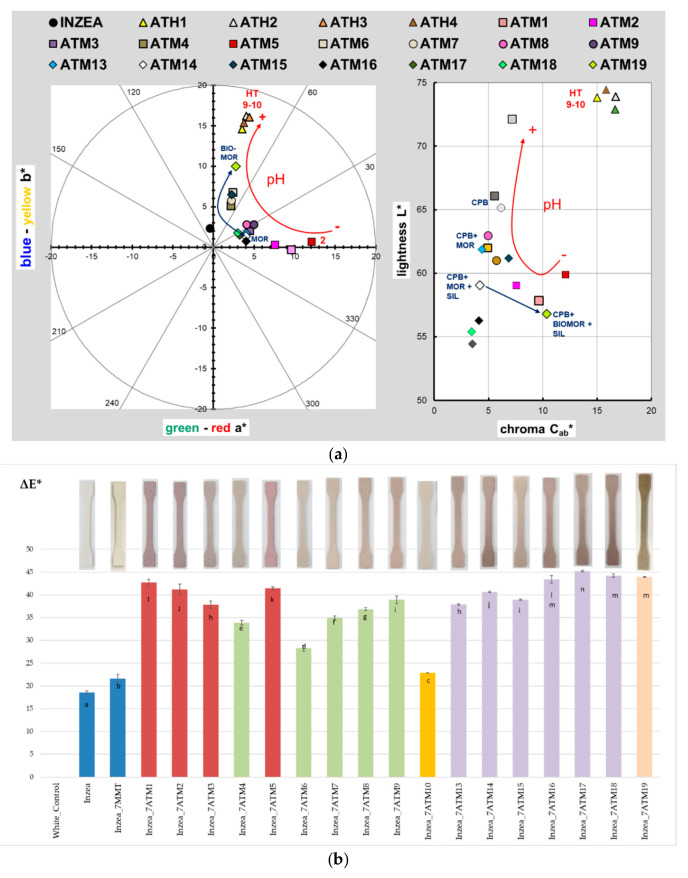
(**a**) CIE-a*b* diagram of the chromatic variation of INZEA-based bionanocomposites containing the most significative ATM and ATH nanofillers with pH. (**b**) Total colour difference of INZEA-based bionanocomposites obtained with MMT-based PDNPs.

Colour is one of the most important properties reflecting the appearance and the final aspect of polymer samples for different aesthetic applications, being the final quality and success of the coloured products determined by the market acceptance. The visual images of the produced ATM-based biopolymer samples are shown in Figure 4. In order to summarize the colour evaluation performed on the nanobiocomposites, Figure 4 also represents the CIE-a*b* diagram of the chromatic variation of INZEA-based bionanocomposites containing the most significative ATM nanofillers as well as total colour difference ∆E* values.

The neat biopolymer was characterized by a high lightness value (81.6 ± 0.3) but the addition of MMT/ATM nanofillers produced a variation in CIELAB colour parameters in the INZEA-based systems (Figure 4). The addition of MMT into INZEA (L* = 79.5 ± 0.7) produced a significant reduction in L* value compared to that obtained for the neat biopolymer due to the intrinsic colour of this nanoclay [2]. The incorporation of different PDNPs in the INZEA-based bionanocomposites, obtained by applying different pH conditions during the synthesis process (Table 1, Figure 1), induced a variation in colour parameters and ΔE* values in the final INZEA_ATM samples (Figure 4) in accordance with the observed colour changes discussed for the hybrid ACN/nanoclay systems.

The presence of nanofillers with initial acid pH synthesis values determined a reddish colour with pink shades in the bionanocomposites and high chromatic hues (Figure 4, positive value of a*) [55,56], obtaining the higher a* value (12.07 ± 0.10) for INZEA_7ATM5 (added with ATM 5 nanofiller which was synthetized at the lower pH = 2). A similar behaviour was observed also for INZEA_7ATM1 (a* = 9.62 ± 0.22). As it has been discussed in Section 3.2, ACNs at acid pH values were stabilized as the flavylium cation form and so intense reds were expected thanks to the extended conjugation between the two aromatic fragments that allows the absorption of visible light with a variable wavelength ranging from 480–550 cm^−1^, depending on the substituents of the rings [23,57,58].

In the presence of nanofillers with neutral to basic pH synthesis values, the colour of INZEA-based bionanocomposites (INZEA_ATM4, INZEA_ATM6 and INZEA_ATM10) drastically changed assuming a greyish and pearl grey colour (Figure 4). The lower a* value was obtained for INZEA_7ATM10 (1.41 ± 0.01) which showed the clearest and most similar colour to that of neat INZEA (light biscuit colour) and the lowest ΔE* value. This behaviour was related to the pH value applied during the nanofiller synthesis without the presence of any modifier (nanopigment ATM10 in Table 1), being ACNs a mixture of neutral and anionic bases slowly evolving into a mixture of hemiketal and chalcones [58].

The modification of MMT-based PDNPs with CPB, mordant and silane additives at different weight amounts (ATM7-ATM9, Table 1) did not induced relevant variations in terms of colour parameters in INZEA-based bionanocomposites (INZEA_7ATM7, INZEA_7ATM8 and INZEA_7ATM9, Figure 4) when the initial pH values of the nanofillers were maintained in the same range (pH = 6–7); according to ACN configurations predominantly due by nucleophilic attack by water. However, the application of basic pH values (9–10) and the addition of CPB, commercial mordant and silane additives at different contents during the synthesis of MMT-based PDNPs (ATM13-ATM18, Table 1) induced a sensible variation in colour parameters and ΔE* values, respect to the nanofillers synthetized at pH = 6–7; showing polymer samples a light greyish colour (Figure 4). In addition, polymer samples containing ATM16–18 nanopigments showed darker and less chromatic colours than polymer samples without the added modifiers at the same pH level (added with nanopigment ATM10). At these high pH values, ANC molecules regain intense colours in the range of blues, greens and yellows, thanks to the predominance of neutral or anionic conformations with strong conjugation. The observed colour changes in PDNPs and the corresponding bathochromic effects were principally caused by structural transformations of ACNs at different pH values, as it has been previously discussed [59]. Similar observations were reported in other studies for different anthocyanin-rich plant extracts [56,60,61].

**Figure 5:**
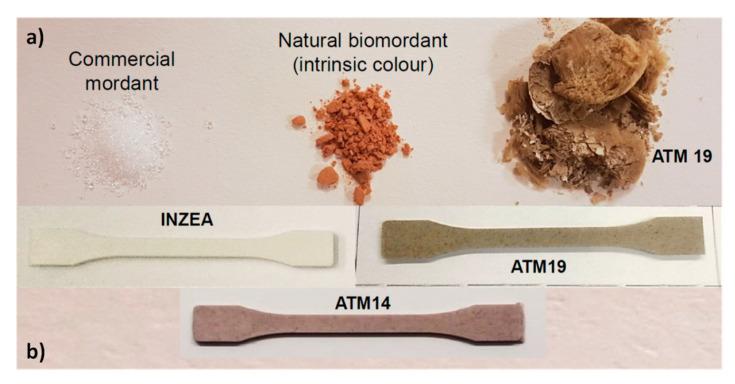
(**a**) Commercial mordant, natural biomordant extracted from pomegranate peel and MMT hybrid nanopigment using PD and the biomordant (ATM19); (**b**) INZEA-based bionanocomposites containing 7 wt% MMT-hybrid nanopigments ATM19 and ATM14.

The incorporation of the natural biomordant extracted from pomegranate peels resulted in a different colour for the resulting nanofiller ATM19 compared to the commercial mordant and equivalent nanofiller ATM14, as previously discussed. This variation influenced also the colour and visual appearance of the developed polymer materials (Figure 5), showing INZEA_7ATM19 a brownish hue compared to INZEA_7ATM14 as a consequence of the natural colour of the pomegranate peel biomordant. So, the use of a different mordant modifier (commercial or natural) induced a radical variation in CIELAB colour parameters (Figure 4). A decrease in a* value was observed with the addition of the natural biomordant (3.98 ± 0.01 for INZEA_7ATM14 vs. 2.75 ± 0.01 for INZEA_7ATM19). In addition, b* values were significantly increased with ATM19 addition compared to ATM14 (1.33 ± 0.06 for INZEA_7ATM14 vs. 9.99 ± 0.01 for INZEA_7ATM19), contributing to a great extent in modifying the ΔE* values and showing the polymer sample loaded with ATM19 a mottled brown colour (Figure 5).

**Figure 6:**
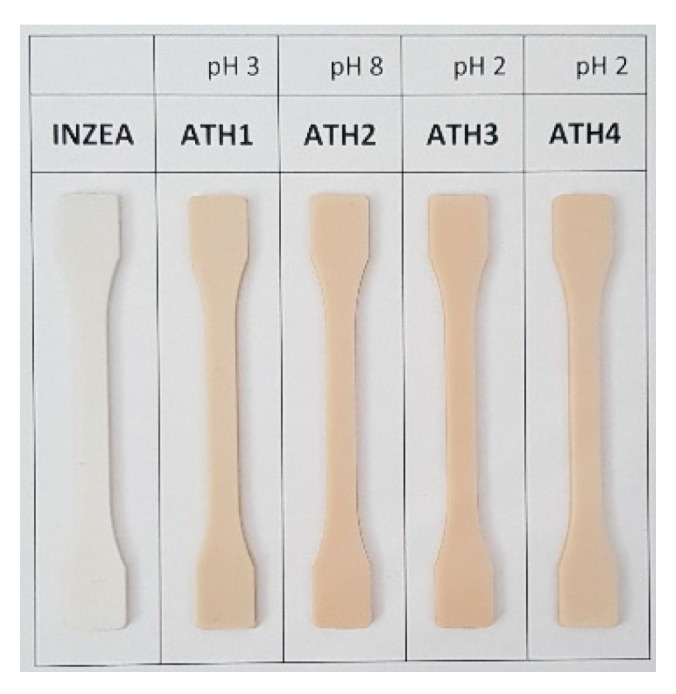
INZEA-based bionanocomposites obtained with HT-based PDNPs.

Regarding HT-based polymer samples (Figure 6), the addition of HT to INZEA (INZEA_7HT) produced an increase in L* value (86.2 ± 0.2) compared to that registered for the neat biopolymer (81.6 ± 0.3) [2]. This phenomenon was due to the HT powder intrinsic colour which modified the final aesthetic appearance of the bionanocomposite sample. The incorporation of the different developed ATH nanofillers into INZEA produced similar CIELAB colour parameters (Figure 4a) and a comparable increase in ΔE* values in the INZEA_7ATH bionanocomposites (Table 4). In conclusion, INZEA-based bionanocomposites did not show significant colour changes with pH modification, acting HT as a buffer and performing a chemical protection not allowing the pH affecting the natural dye, getting brownish/yellowish and lighter colours.

**Figure 7:**
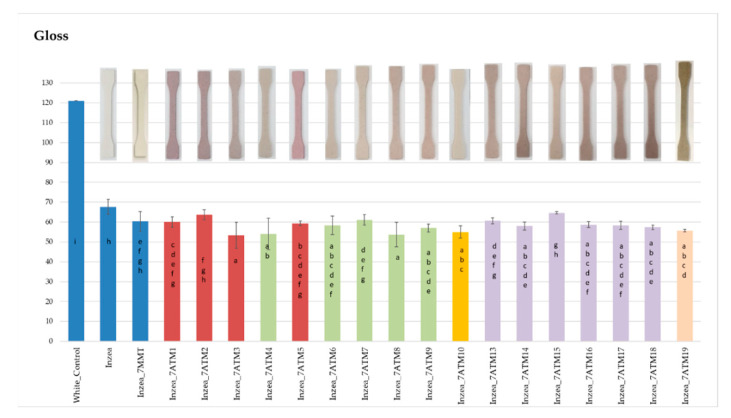
Gloss values obtained for INZEA-based bionanocomposites containing 7 wt% of the most significant ATM nanofillers (Different superscripts in the same column for each formulation indicate statistically significant different values (p < 0.05)).

The presence of unmodified MMT and HT as well as ATM and ATH nanofillers produced a reduction in gloss values in the bionanocomposites (Figure 7 and Table 4), which was also affected by the hybrid nanopigment synthesis conditions. INZEA-based bionanocomposites containing ATM2 (pH 4–5, without modifiers) and ATM15 (pH 9–10, added with the three modifiers CPB, MOR, and SIL) presented the highest gloss values.

**Figure 8:**
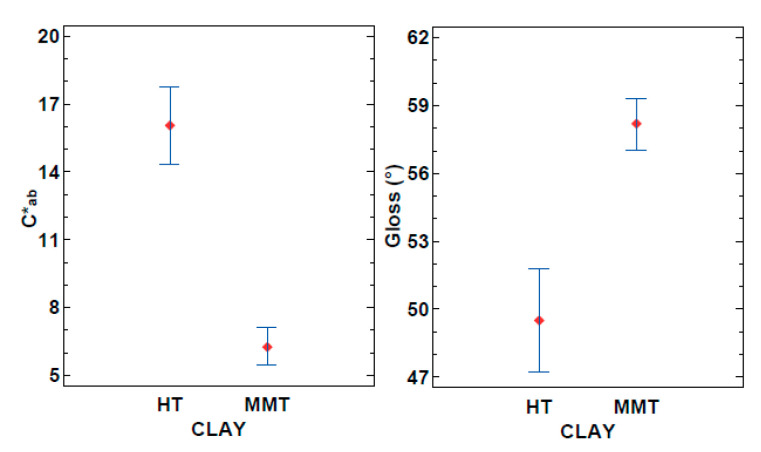
LSD turkey means plot (95 confidence intervals) from ANOVA test with CLAY factor and C_ab_* and Gloss (°) dependent variables.

Finally, statistically significant differences between the nanoclay structure used for PDNPs synthesis and the final optical properties of the bionanocomposites in terms of C_ab_* and gloss values were obtained (*p* < 0.05). In this sense, HT-based samples showed higher chromatic and lower gloss values compared to MMT-based hybrid pigments (Figure 8).

#### 3.3.2. Thermal Stability

**Figure 9:**
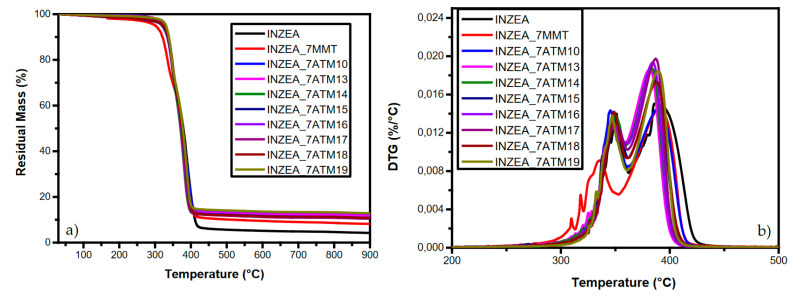

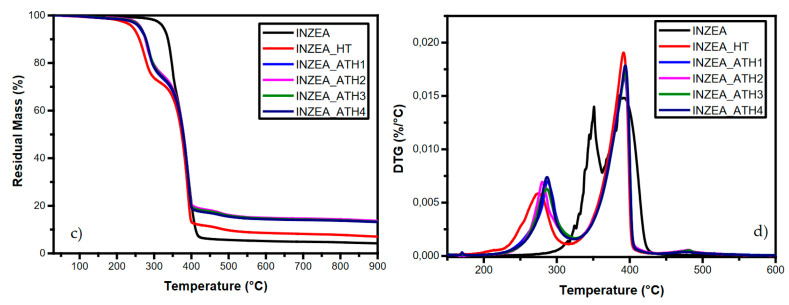
TG and DTG profiles for INZEA-based bionanocomposites containing MMT (**a**,**b**) and HT (**c**,**d**) functionalized PDNPs.

The observed behaviour for PDNPs greatly influenced the thermal properties of INZEA-based systems. The curves related to mass loss and derivative weight loss for some of the bionanocomposites added with 7 wt% of the MMT-based nanopigments are shown in Figure 9a,b. As it has been reported [2], a double degradation peak can be found for the INZEA neat matrix, with two main steps centred at 350 and 400 °C, which could match with the possible degradation temperatures of PLA and poly(butylene succinate) (PBS) polyesters, respectively. In addition, unmodified INZEA matrix maintained a residual mass of ca. 5 wt% at 900 °C, which was increased when the nanofillers were introduced. This behaviour is in accordance with the possible presence of an inorganic filler in the formulation of the commercial product, as it has been reported in other work [62].

A deep analysis of the DTG curves for the bionanocomposites containing neat MMT or HT and PDNPs (Figure 9) clearly indicated that, while no substantial variations were noted in the case of MMT-based systems, the thermal stability of the overall blend was strongly affected with the addition of HT and ATH nanofillers. Specifically, while the maximum degradation rate remained substantially unaffected at higher temperatures, the weight loss rate of the less stable components was shifted from 350 °C for neat INZEA to 287 and 276 °C, respectively, for INZEA_HT and INZEA_HT1-INZEA_HT4 samples (Figure 9c,d). The obtained degradation path for bionanocomposites, differing from that of the neat matrix, can be rationalized by considering the organic nature of the nanopigments which were indeed responsible of the decrease in thermal stability of the polyester matrix. In this sense, both unmodified nanoclays could catalyse hydrolysis reactions, contributing MMT platelets with–OH moieties present on the surface whereas the presence of moisture in the interlayer spacing of hydrotalcites could affect the thermodegradative pattern of the corresponding bionanocomposites.

#### 3.3.3. Tensile Properties

**Figure 10:**
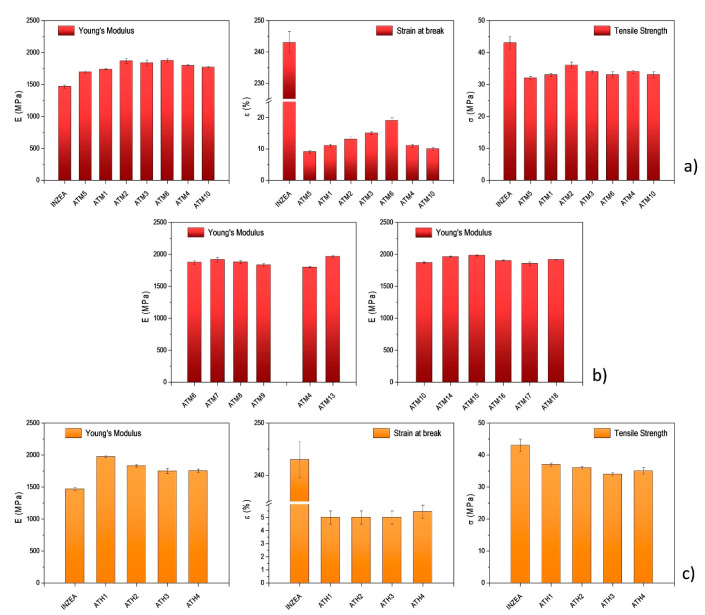
Results for tensile tests of INZEA_MMT PDNPs as a function of pH (**a**) and different silane/surfactant/mordant ratios (**b**). INZEA_HT PDNPs (**c**).

The tensile results obtained for bionanocomposites containing 7 wt% of MMT-based nanopigments synthetized at different pH values are shown in Figure 10a. The Young modulus (E) values of all polymer samples were higher than that obtained for the neat matrix, showing an increasing trend for E values moving from acid pH values to neutral ones. After that, the elastic modulus decreased again moving from neutral towards alkaline pH values. In particular, INZEA containing ATM6 showed an elastic modulus value of 1874 ± 65 MPa, with an increase of 28% compared to neat INZEA and 41% compared to the bionanocomposite with the unmodified MMT [2]. A similar trend was also found for the strain at break: moving from the lower pH to neutral, the elongation at break increased progressively and decreased again for pH values higher than neutral. However, the tensile stress values did not show any significant changes. The synthesis treatment of the nanofillers at different pH values also affected the tensile properties of the bionanocomposites. In general, the nanofillers synthesis process was proved to be effective for improving the mechanical properties of the polymer samples, overpassing the reference values of the bionanocomposite with unmodified MMT (elastic modulus = 1331 ± 28 MPa, tensile strength = 34 ± 1 MPa, elongation at break = 7% ± 1%) [2]. In particular, it should be noted that samples with nanofillers synthetized at slightly acid or neutral pH values showed the best mechanical properties. This result can be explained by considering that a slightly acid environment could favour the compatibility between the polymer matrix and the PDNP filler [63] compared to an alkaline one, but excessively acid pH values could induce a corrosion phenomenon that could damage the bionanocomposites, worsening their mechanical properties [64].

The effect of the added modifiers to bionanocomposites containing MMT-based nanopigments is shown in Figure 10b. ATM7 nanofiller included the presence of the anionic surfactant CPB at 5 wt%. The introduction of CPB and the inorganic commercial mordant at 5 wt% was considered in ATM8, while ATM9 included also the silane at 1 wt% (Table 2). On the other hand, the nanopigments ATM13-ATM19 included different concentrations of the 3 modifiers. The tensile properties of polymer samples showed slight variations in the characteristic properties values. Among the samples synthetized at pH 6–7, only INZEA added with ATM7 showed an increase in Young’s modulus up to 1915 MPa, while the tensile strength improved, albeit slightly, for all the polymer formulations. The strain at break of these samples was approximately half reduced compared to INZEA_7ATM6. INZEA_7ATM13, the formulation including the nanofiller ATM13 synthetized at pH 7–8, showed an improvement in the elastic modulus up to 1965 MPa, which corresponded to negligible variations in σ and ε. In general, the use of the nanofillers was intended to improve the colour characteristics of the materials without compromising, and possibly improving, the mechanical properties. This purpose can be considered achieved, even if with limited improvement, as there were no significant changes in the mechanical properties such as to compromise their use. On the contrary, slight improvements were obtained in terms of elastic modulus which confirmed the applicability of the developed bionanocomposite materials. On the other hand, it should be noted that the added quantities of the nanofillers were quite small to show an evident effect on mechanical properties. The surfactant was used in order to improve the dispersion of the pigment on the nanofiller and to give a more intense colour, but it could also have a beneficial effect in preventing the agglomeration of the nanometric MMT into the bionanocomposite. In the face of evident chromatic variations between the nanopigments synthetized at pH 9–10, no significant variations in mechanical properties were observed in the polymer samples both in terms of σ and ε values; and the only noteworthy result was a slight increase in the elastic modulus, rising up to 1983 MPa for INZEA_7ATM15 with an increase of 12% compared to INZEA_7ATM10, synthetized without any modifier addition. Regarding the mechanical differences between bionanocomposites incorporated with ATM14 and ATM19 nanofillers (including the synthetic mordant and biomordant, respectively), limited changes were observed in terms of tensile strength and deformation at break: these values were almost similar for the two systems and equal, respectively, to σ = 35 ± 2 MPa and ε = 9±1% in the case of INZEA_7ATM19, while values of σ = 34 ± 2 MPa and ε = 10 ± 1% were measured in the case of INZEA_7ATM14 formulation. A slight difference was noted in the values of elastic moduli, respectively measured as E = 1960 ± 29 MPa and E = 1719 ± 95 MPa for INZEA_7ATM14 and INZEA_7ATM19, respectively, that did not compromise the stiffness of the equivalent formulation.

The mechanical characteristics of the bionanocomposites filled with HT-based nanopigments were influenced by the buffer behaviour of the nanofiller which limited colour variations, bringing all nanofiller formulations to alkaline pH values after the synthesis process (Table 2), and also altering the interaction between the biopolymer matrix and the nanoclay. These reactions had an effect on the ion exchange capacity of hydrotalcite and consequently on the matrix/charge compatibility [65]. According to E values, INZEA_HT1 sample showed the most positive effect on the matrix/modified nanofiller compatibility (Figure 10c). Although all ATH nanofillers produced a general increase in the elastic modulus, which was more relevant (34%) for ATH1 (1973 MPa) and to a slightly lesser extent (25%) for ATH2 (1830 MPa) compared to the biopolymer matrix, a limited effect on deformation at break was observed resulting in values which were maintained around 5% for all HT-based polymer samples. Due to the reduced deformability, although the σ-ε curves of the bionanocomposites had a faster rise, the samples achieved lower strength values compared to the matrix. Bionanocomposites with the same deformation and higher Young modulus also showed higher tensile stresses (INZEA_HT1 showed, in this case, the best performance with 37 MPa followed by INZEA_HT2 with 36 MPa, and slightly lower values for INZEA_HT3 and INZEA_HT4).

### 3.4. LCA

In the pomegranate pigment extraction model, some assumptions were made to better estimate the waste origin of the biomass. Firstly, since pomegranate fruit was not available in software libraries, its impact was modelled using the literature data taking into consideration the management of the field, the growth of the fruit and the harvesting operation. Additionally, since only pomegranate waste was used for pigment extraction, a resource allocation was performed allowing a partial attribution of all resources used to produce the fruits to the waste. The criteria used for allocation was the mass ratio between the fruit waste and the overall produced fruit. Additionally, some hypothesis on nanofillers functionalization were done regarding the selection of the used chemicals, since the surfactant cetylpyridinium bromide (CPB) and the mordant agent (aluminium potassium sulphate dodecahydrate) were not present in the libraries, they were modelled using the literature data.

A preliminary comparison of functionalized MMT and HT-based nanofillers was performed (ATM14 and ATH4 nanofillers were taken as a reference and considered for this purpose). The results obtained in terms of environmental impacts achieved with ReCiPe 2016 (H) at midpoint are reported in Table 5. MMT and HT hybrid nanofillers showed a very similar environmental profile. All environmental category indicators analysed with ReCiPe 2016 method were similar for the two functionalized nanofillers, showing ATH4 always a slightly higher value. For example, for ATH4 the global warming potential, mineral resources scarcity and fossil resources scarcity were 4.5%, 3.9% and 4.65% higher, respectively, compared to ATM14. These differences can be mainly attributed to a different energy consumption in the functionalized nanofiller production. In fact, for HT an initial calcination phase absorbing an extra energy consumption was required, while this step was absent in the MMT functionalization. Therefore, it is possible to assert that both hybrid nanopigments were quite similar in terms of environmental performance, showing a slightly better general behaviour for the MMT-based hybrid nanopigment.

In order to verify the effect of introducing a biomordant obtained from pomegranate peels on the environmental impact of the functionalization procedures, the comparison between ATM14 and ATM19 hybrid nanopigments was considered. Figure S1 reports the process tree relative to the LCA model of the MMT functionalization process, while Table 6 reports the results of the assessment made between MMT functionalized by using the biomordant and the commercial mordant. The process tree evidenced the different processing steps modelled for the production process and the red lines gave an immediate visibility about the contribution of each single step in the overall environmental weight. It is evident that the boxes related to energy had the highest weight. Both nanofillers had a very similar environmental profile; nevertheless, the process considering the bio-based mordant showed slightly higher environmental indicators (Table 6) for all categories.

The differences found in terms of environmental profile for nanofillers was reflected also on the environmental profile of the biopolymer formulations containing the polyester fraction. Table 7 reports the results obtained for the characterization of the bionanocomposites production using INZEA filled with ATM14 and ATM19 nanopigments. By comparing the results of the environmental categories indicators in Table 6 and Table 7 it is evident that the nanofillers had a marginal contribution to the environmental profile of the polymer materials. Considering that the contribution of INZEA polymer and the compounding is constant in both analysed materials, the two hybrid nanopigments had a similar effect on the environmental profile of the resulting bionanocomposites, confirming that INZEA_ATM 19 had a slightly higher environmental category indicators profile.

Finally, an analysis focusing on the performance of the nanofillers and the relative polymer materials on GHG emission was also performed using the impact assessment method IPCC 20yr. Table 8 reports the obtained results expressed in terms of kg of CO_2_ equivalents per kg of product. As it can be observed, both nanofillers and nanobiocomposites showed very similar GHG emissions profile, confirming the results achieved in the other indicators analysed with the ReCiPe method, with a slightly better performance for the processes including the commercial mordant.

The results of this LCA study evidenced how the circular bioeconomy approach can be achieved through sustainable food waste management. In parallel, future developments on food waste management are expected to capitalise on the multi-functionality of products, boundary and allocation in a circular system, and trade-off between food waste and resources [66,67]. Additionally, circular bioeconomy is one of the important principles of economic policies and bioplastics produced using waste substrates fit perfectly in this concept. In addition to the production using waste substrates, sustainability assessment has to be carried out with respect to environment [68]. However, more comparative studies are required for the entire production process, by considering waste streams and recycling at each step. It should be also considered that studies analysing the feasibility at industrial scale of these procedures are still missing [69]. Relevant aspects to be further explored for a comprehensive techno-economic analysis of the biorefinery are feedstock availability and logistics, and market price of value-added products.

## 4. Conclusions

Novel polyester-based bionanocomposites incorporating 7 wt% of anthocyanin pomegranate hybrid nanopigments (PDNPs) were successfully developed and characterized in this work. PDNPs were synthetized by using a natural pomegranate dye (PD) intercalated into MMT and HT nanoclays, preserving the dye thermal stability. All HT-based PDNPs showed a similar yellowish colour due to a buffer effect of HT whereas different chromatic hues were obtained for MMT-based nanopigments depending on pH. Reddish nanopigments were obtained at acid pH values suggesting a good stabilization of PD in its flavylium cation form whereas yellowish and bluish hues were observed at higher pH values as a consequence of structural transformations of ACNs, obtaining a wide colour gamut of bionanocomposites by using the proposed synthesis methods. The use of a pomegranate peel biomordant in the synthesis of MMT-based nanopigments induced some colour differences due to its intrinsic colouring properties. Moreover, some reinforcement effect was also observed for MMT-based nanopigments in terms of elastic modulus, suggesting the potential of the obtained biomaterials as coloured reinforced bionanocomposites for different applications, such as automotive and construction. The processes used and the biomaterials obtained in this work were also validated in terms of life cycle assessment contributing to the circular bioeconomy approach. Further work will be needed to evaluate functional (antimicrobial activity, disintegrability, recyclability) and environmental profiles of the obtained biomaterials at a larger industrial scale.

## Figures and Tables

**Table 1:** Experimental conditions used for the synthesis of MMT and HT-based pomegranate hybrid nanopigments.

Code	Nanoclay	Initial pH	Final pH	Dye (g L^−1^)	Surfactant (wt%) *	Mordant (wt%)	Silane (wt%)
ATM1	MMT	[3,4]	5.5	0.56	-	-	-
ATM2	MMT	[4,5]	6.3	0.56	-	-	-
ATM3	MMT	[5,6]	7.1	0.56	-	-	-
ATM4	MMT	[7,8]	8.5	0.56	-	-	-
ATM5	MMT	2.0	3.7	0.56	-	-	-
ATM6	MMT	[6,7]	8.3	0.56	-	-	-
ATM7	MMT	[6,7]	7.9	0.56	5	-	-
ATM8	MMT	[6,7]	7.1	0.56	5	5	-
ATM9	MMT	[6,7]	6.8	0.56	5	5	1
ATM10	MMT	[9,10]	10.9	0.56	-	-	-
ATM11	MMT	[10,11]	11.5	0.56	-	-	-
ATM12	MMT	[11,12]	11.9	0.56	-	-	-
ATM13	MMT	[7,8]	9.3	1.11	10	10	2
ATM14	MMT	[9,10]	9.8	1.11	10	10	2
ATM15	MMT	[9,10]	9.7	1.11	10	5	1
ATM16	MMT	[9,10]	9.8	1.11	10	10	1
ATM17	MMT	[9,10]	9.7	1.76	10	5	-
ATM18	MMT	[9,10]	9.8	1.11	10	5	2
ATM19	MMT	[9,10]	7	1.11	10	10 **	2
ATH1	HT	3.1	8.8	0.56	-	-	-
ATH2	HT	8.4	11.5	0.56	-	-	-
ATH3	HT	2.0	9.6	0.56	-	-	-
ATH4	HT	1.6	9.1	0.56	5	5	-

* Cetylpyridinium bromide (CPB) was used for MMT and sodium dodecyl sulphate (SDS) for HT. ** Pomegranate peel biomordant.

**Table 2:** Content and UHPLC-DAD-MS conditions of major ACNs found in pomegranate pigment. Mean ± SD, *n* = 3.

Compound	R_1_	R_2_	R_3_	R_4_	R_5_	RT (min)	MRM	Cone (V)	Collision (V)	Concentration (wt%)
Delphinidin-3,5-Diglucoside	OH	OH	OH	Glu *	Glu *	2.47	627.04 > 465.02	10	20	10.581 ± 0.032
Cyanidin-3,5-diglucoside	OH	OH	H	Glu *	Glu *	2.81	611.03 > 449.19	64	18	12.819 ± 0.014
Pelargonidin-3,5-diglucoside	H	OH	H	Glu *	Glu *	3.08	595.03 > 433.06	10	18	0.796 ± 0.007
Delphinidin-3-glucoside	OH	OH	OH	Glu *	OH	3.33	464.89 > 303.04	34	22	1.440 ± 0.020
Cyanidin-3-glucoside	OH	OH	H	Glu *	OH	3.67	448.89 > 137.02	2	54	1.860 ± 0.071
Pelargonidin-3-glucoside	H	OH	H	Glu *	OH	3.99	432.97 > 271.05	4	22	0.230 ± 0.020

* Glu: glucoside; Flavylium cationic form.

**Table 3:** Additional experiments performed with different biomordant, surfactant and silane concentrations in ATM19.

Code	Biomordant (wt%)	Surfactant (CPB, wt%)	Silane (wt%)
ATM19_2	1	2	1
ATM19_3	1	1	2
ATM19_4	1	5	2
ATM19_6	5	5	2
ATM19_7	2	2	2
ATM19_9	2	1	5
ATM19_10	2	1	1
ATM19_11	5	2	5

**Table 4:** CIELAB parameters for INZEA-based bionanocomposites with HT and ATH nanopigments (mean ± SD, *n* = 3).

Formulations	L*	a*	b*	∆E*	Gloss (°)	C_ab_*
**White Control**	99.5 ± 0.1 ^a^	−0.08 ± 0.01 ^a^	−0.08 ± 0.01 ^a^	-	121 ± 2 ^d^	0.02 ± 0.01 ^a^
**INZEA**	81.6 ± 0.3 ^b^	0.65 ± 0.05 ^a^	5.11 ± 0.04 ^c^	18.6 ± 0.3 ^b^	68 ± 3 ^c^	5.16 ± 0.05 ^c^
**INZEA_7HT**	86.2 ± 0.2 ^c^	0.14 ± 0.04 ^b^	4.22 ± 0.08 ^b^	13.9 ± 0.2 ^a^	57 ± 1 ^b^	4.22 ± 0.08 ^b^
**INZEA_7ATH1**	73.8 ± 1.4 ^d^	3.53 ± 0.30 ^c^	14.60 ± 0.20 ^c^	29.8 ± 1.1 ^c^	50 ± 4 ^a^	15.00 ± 0.14 ^d^
**INZEA_7ATH2**	73.9 ± 1.3 ^d^	4.07 ± 0.33 ^d,e^	16.21 ± 0.42 ^e^	30.6 ± 1.0 ^c^	48 ± 6 ^a^	16.70 ± 0.30 ^f^
**INZEA_7ATH3**	72.9 ± 1.6 ^d^	4.50 ± 0.40 ^e^	16.10 ± 0.21 ^e^	31.4 ± 1.5 ^c^	50 ± 6 ^a,b^	16.60 ± 0.30 ^f^
**INZEA_7ATH4**	74.5 ± 1.7 ^d^	3.70 ± 0.41 ^c,d^	15.42 ± 0.40 ^d^	29.7 ± 1.7 ^c^	50 ± 6 ^ab^	15.80 ± 0.40 ^e^

Different superscripts in the same column for each formulation indicate statistically significant different values (*p* < 0.05).

**Table 5:** Comparison of main impact categories involved for MMT and HT-based hybrid nanopigments production (ReCiPe 2016 (H) method at mid-point).

Impact Category	Unit	ATM14	ATH4
Global warming	kg CO_2_ eq.	2.00	2.09
Stratospheric ozone depletion	kg CFC11 eq.	9.74 × 10^−7^	9.93 × 10^−7^
Ionizing radiation	kBq Co-60 eq.	1.07	1.11
Ozone formation, human health	kg NO_x_ eq.	5.81 × 10^−3^	6.06 × 10^−3^
Fine particulate matter formation	kg PM 2.5 eq.	4.94 × 10^−3^	5.14 × 10^−3^
Ozone formation, terrestrial ecosystems	kg NO_x_ eq.	5.84 × 10^−3^	6.09 × 10^−3^
Terrestrial acidification	kg SO_2_ eq.	1.25 × 10^−2^	1.30 × 10^−2^
Freshwater eutrophication	kg P eq.	7.62 × 10^−4^	7.95 × 10^−4^
Marine eutrophication	kg N eq.	7.13 × 10^−5^	7.25 × 10^−5^
Terrestrial ecotoxicity	kg 1,4-DCB	4.73	4.92
Freshwater ecotoxicity	kg 1,4-DCB	1.71 × 10^−1^	1.79 × 10^−1^
Marine ecotoxicity	kg 1,4-DCB	2.15 × 10^−1^	2.24 × 10^−1^
Human carcinogenic toxicity	kg 1,4-DCB	8.62 × 10^−2^	8.99 × 10^−2^
Human non-carcinogenic toxicity	kg 1,4-DCB	1.42	1.48
Land use	m^2^a crop eq.	6.17 × 10^−2^	6.23 × 10^−2^
Mineral resource scarcity	kg Cu eq.	4.10 × 10^−3^	4.26 × 10^−3^
Fossil resource scarcity	kg oil eq.	4.73 × 10^−1^	4.95 × 10^−1^
Water consumption	m^3^	1.93 × 10^−2^	1.94 × 10^−2^

**Table 6:** Characterization results for the production of ATM14 (traditional mordant) and ATM19 (biomordant) nanopigments.

Impact Category	Unit	ATM14	ATM19
Global warming	kg CO_2_ eq.	2.00	2.03
Stratospheric ozone depletion	kg CFC11 eq.	9.74 × 10^−7^	1.04 × 10^−6^
Ionizing radiation	kBq Co-60 eq.	1.07	1.08
Ozone formation, human health	kg NO_x_ eq.	5.81 × 10^−3^	5.90 × 10^−3^
Fine particulate matter formation	kg PM2.5 eq.	4.94 × 10^−3^	4.99 × 10^−3^
Ozone formation, terrestrial ecosystems	kg NO_x_ eq.	5.84 × 10^−3^	5.93 × 10^−3^
Terrestrial acidification	kg SO_2_ eq.	1.25 × 10^−2^	1.26 × 10^−2^
Freshwater eutrophication	kg P eq.	7.62 × 10^−4^	7.70 × 10^−4^
Marine eutrophication	kg N eq.	7.13 × 10^−5^	7.67 × 10^−5^
Terrestrial ecotoxicity	kg 1,4-DCB	4.73	4.78
Freshwater ecotoxicity	kg 1,4-DCB	1.71 × 10^−1^	1.73 × 10^−1^
Marine ecotoxicity	kg 1,4-DCB	2.15 × 10^−1^	2.17 × 10^−1^
Human carcinogenic toxicity	kg 1,4-DCB	8.62 × 10^−2^	8.72 × 10^−2^
Human non-carcinogenic toxicity	kg 1,4-DCB	1.42	1.43
Land use	m2a crop eq.	6.17 × 10^−2^	6.74 × 10^−2^
Mineral resource scarcity	kg Cu eq.	4.10 × 10^−3^	4.14 × 10^−3^
Fossil resource scarcity	kg oil eq.	4.73 × 10^−1^	4.78 × 10^−1^
Water consumption	m^3^	1.93 × 10^−2^	2.16 × 10^−2^

**Table 7:** Impact categories involved in the development of INZEA-based bionanocomposites containing 7 wt% of ATM14 and ATM19 pomegranate nanohybrids.

Impact Category	Unit	INZEA_ATM14	INZEA_ATM19
Global warming	kg CO_2_ eq.	2.04 × 10^+2^	2.06 × 10^+2^
Stratospheric ozone depletion	kg CFC11 eq.	1.03 × 10^−4^	1.09 × 10^−4^
Ionizing radiation	kBq Co-60 eq.	1.07 × 10^+2^	1.08 × 10^+2^
Ozone formation, human health	kg NO_x_ eq.	5.88 × 10^−1^	5.97 × 10^−1^
Fine particulate matter formation	kg PM2.5 eq.	5.00 × 10^−1^	5.05 × 10^−1^
Ozone formation, terrestrial ecosystems	kg NO_x_ eq.	5.91 × 10^−1^	6.00 × 10^−1^
Terrestrial acidification	kg SO_2_ eq.	1.26	1.28
Freshwater eutrophication	kg P eq.	7.73 × 10^−2^	7.81 × 10^−2^
Marine eutrophication	kg N eq.	8.67 × 10^−3^	9.21 × 10^−3^
Terrestrial ecotoxicity	kg 1,4-DCB	4.80 × 10^+2^	4.85 × 10^+2^
Freshwater ecotoxicity	kg 1,4-DCB	1.73 × 10^+1^	1.75 × 10^+1^
Marine ecotoxicity	kg 1,4-DCB	2.17 × 10^+1^	2.20 × 10^+1^
Human carcinogenic toxicity	kg 1,4-DCB	8.74	8.84
Human non-carcinogenic toxicity	kg 1,4-DCB	1.47 × 10^+2^	1.48 × 10^+1^
Land use	m^2^a crop eq.	6.90	7.47
Mineral resource scarcity	kg Cu eq.	4.17 × 10^−1^	4.21 × 10^−1^
Fossil resource scarcity	kg oil eq.	4.85 × 10^+1^	4.91 × 10^+1^
Water consumption	m^3^	1.98	2.20

**Table 8:** IPCC 20yr results for the two nanofillers (ATM14 and ATM19) and the related polyester materials.

Material	IPCC GWP 20yr (kg CO_2_ Eq/Kg)
ATM 14	2.27
ATM 19	2.31
INZEA_ATM14	231
INZEA_ATM 19	234

## Data Availability

The data presented in this study are available on request from the corresponding author.

## Supplementary Materials

The following are available online at https://www.mdpi.com/article/10.3390/polym13121966/s1, Figure S1: MMT-based PDNPs process tree and details of the three sections: pomegranate dye extraction (a), MMT functionalization with surfactant and mordant (b) and nanohybrid preparation (c).
