# Supplementary material for: Anthocyanin Hybrid Nanopigments from Pomegranate Waste: Colour, Thermomechanical Stability and Environmental Impact of Polyester-Based Bionanocomposites
Source: Polymers (Basel). 2021 Jun 14;13(12):1966. doi: 10.3390/polym13121966 (PMC8232300; doi:10.3390/polym13121966)
Supplement: Supplementary file 1 [file polymers-13-01966-s001.zip › polymers-1254662-SI.pdf]

Anthocyanin hybrid nanopigments from pomegranate waste: colour, thermomechanical stability and environmental impact of polyester-based bionanocomposites

Bàrbara Micó-Vicent[1,2], Marina Ramos [3], Valentin Viqueira [1], Francesca Luzi [4], Franco Dominici [4], Andrea Terenzi [4], Etienne Maron[5], Mahmoud Hamzaoui[5], Stephane Kohnen[5], Luigi Torre [4], Alfonso Jiménez [3], Debora Puglia [4],* and María Carmen Garrigós [3],*

[1] Colour and Vision Group, University of Alicante, San Vicente del Raspeig, ES-03690, Alicante, Spain; barbara.mico@ua.es (B.M.); valentin.viqueira@ua.es (V.V.)

[2] Department of Appl. Stat. & Operat. Research, & Qual., Universitat Politècnica de València, ES-03801, Valencia, Spain; barbara.mico@gmail.com (B.M.)

[3] Department of Analytical Chemistry, Nutrition & Food Sciences, University of Alicante, San Vicente del Raspeig, ES-03690, Alicante, Spain; marina.ramos@ua.es (M.R.); alfjimenez@ua.es (A.J.)

[4] Department of Civil and Environmental Engineering, University of Perugia, 05100, Terni, Italy; francesca.luzi@unipg.it (F.L.); francodominici1@gmail.com (F.D.); andrea.terenzi@unipg.it (A.T.); luigi.torre@unipg.it (L.T.)

[5] Biomass Valorisation Platform, Celabor scrl, Avenue du Parc 38, 4650 Herve, Belgium; Etienne.Maron@celabor.be (E.M.); Mahmoud.Hamzaoui@celabor.be (M.H.); Stephane.Kohnen@celabor.be (S.K.)

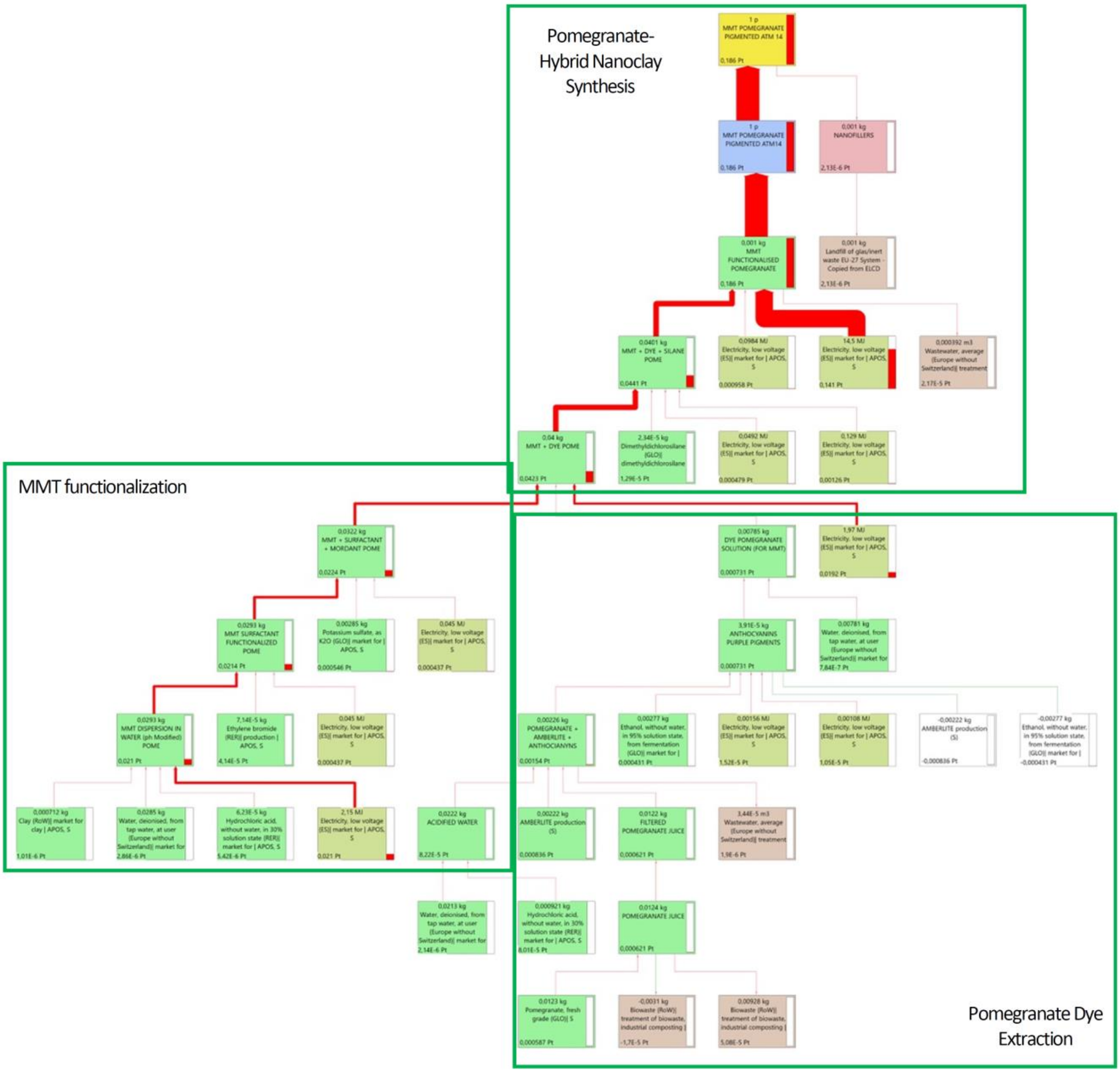

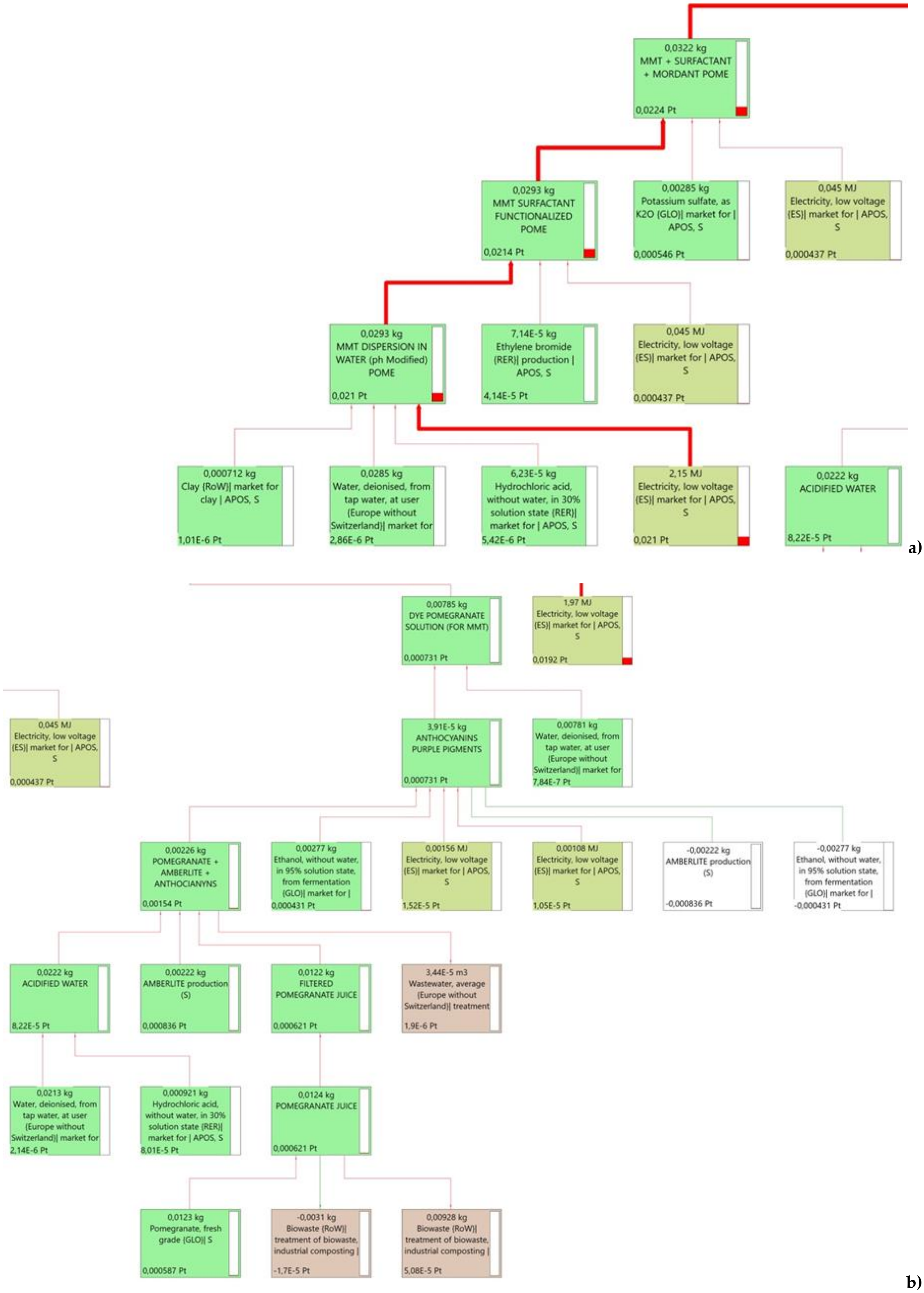

0,0322 kg
MMT + SURFACTANT + MORDANT POME
0,0224 Pt
0,0293 kg
MMT SURFACTANT FUNCTIONALIZED POME
0,0214 Pt
0,00285 kg
Potassium sulfate, as K2O {GLO}| market for | APOS, S
0,000546 Pt
0,045 MJ
Electricity, low voltage {ES}| market for | APOS, S
0,000437 Pt
0,0293 kg
MMT DISPERSION IN WATER (ph Modified) POME
0,021 Pt
7,14E-5 kg
Ethylene bromide {RER}| production | APOS, S
4,14E-5 Pt
0,045 MJ
Electricity, low voltage {ES}| market for | APOS, S
0,000437 Pt
0,000712 kg
Clay {RoW}| market for clay | APOS, S
1,01E-6 Pt
0,0285 kg
Water, deionised, from tap water, at user {Europe without Switzerland}| market for
2,86E-6 Pt
6,23E-5 kg
Hydrochloric acid, without water, in 30% solution state {RER}| market for | APOS, S
5,42E-6 Pt
2,15 MJ
Electricity, low voltage {ES}| market for | APOS, S
0,021 Pt
0,0222 kg
ACIDIFIED WATER
8,22E-5 Pt
a)
0,00785 kg
DYE POMEGRANATE SOLUTION (FOR MMT)
0,000731 Pt
1,97 MJ
Electricity, low voltage {ES}| market for | APOS, S
0,0192 Pt
0,045 MJ
Electricity, low voltage {ES}| market for | APOS, S
0,000437 Pt
3,91E-5 kg
ANTHOCYANINS PURPLE PIGMENTS
0,000731 Pt
0,00781 kg
Water, deionised, from tap water, at user {Europe without Switzerland}| market for
7,84E-7 Pt
0,00226 kg
POMEGRANATE + AMBERLITE + ANTHOCIANYNS
0,00154 Pt
0,00277 kg
Ethanol, without water, in 95% solution state, from fermentation {GLO}| market for |
0,000431 Pt
0,00156 MJ
Electricity, low voltage {ES}| market for | APOS, S
1,52E-5 Pt
0,00108 MJ
Electricity, low voltage {ES}| market for | APOS, S
1,05E-5 Pt
-0,00222 kg
AMBERLITE production (S)
-0,000836 Pt
-0,00277 kg
Ethanol, without water, in 95% solution state, from fermentation {GLO}| market for |
-0,000431 Pt
0,0222 kg
ACIDIFIED WATER
8,22E-5 Pt
0,00222 kg
AMBERLITE production (S)
0,000836 Pt
0,0122 kg
FILTERED POMEGRANATE JUICE
0,000621 Pt
3,44E-5 m3
Wastewater, average {Europe without Switzerland}| treatment
1,9E-6 Pt
0,0213 kg
Water, deionised, from tap water, at user {Europe without Switzerland}| market for
2,14E-6 Pt
0,000921 kg
Hydrochloric acid, without water, in 30% solution state {RER}| market for | APOS, S
8,01E-5 Pt
0,0124 kg
POMEGRANATE JUICE
0,000621 Pt
0,0123 kg
Pomegranate, fresh grade {GLO}| S
0,000587 Pt
-0,0031 kg
Biowaste {RoW}| treatment of biowaste, industrial composting |
-1,7E-5 Pt
0,00928 kg
Biowaste {RoW}| treatment of biowaste, industrial composting |
5,08E-5 Pt
b)

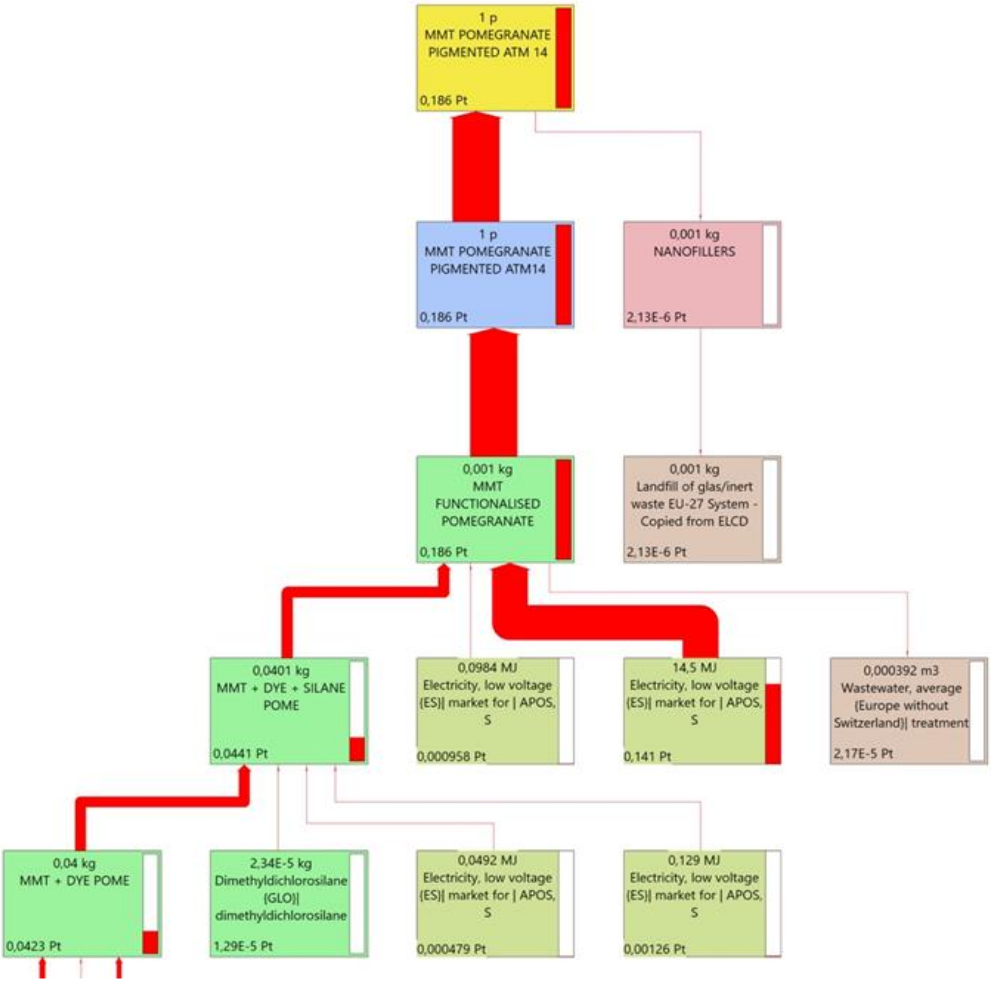

c)

Figure S1. MMT-based PDNPs process tree and details of the three sections: pomegranate dye extraction (a), MMT functionalization with surfactant and mordant (b) and nanohybrid preparation (c).
